# Dynamic Expressions of TIGIT on Splenic T Cells and TIGIT-Mediated Splenic T Cell Dysfunction of Mice With Chronic *Toxoplasma gondii* Infection

**DOI:** 10.3389/fmicb.2021.700892

**Published:** 2021-08-05

**Authors:** Haoran Li, Jing Zhang, Changwei Su, Xiaowei Tian, Xuefang Mei, Zhenchao Zhang, Mingyong Wang, Xiangrui Li, Shuai Wang

**Affiliations:** ^1^Xinxiang Key Laboratory of Pathogenic Biology, Department of Pathogenic Biology, School of Basic Medical Sciences, Xinxiang Medical University, Xinxiang, China; ^2^Xinxiang Key Laboratory of Immunoregulation and Molecular Diagnostics, School of Laboratory Medicine, Xinxiang Medical University, Xinxiang, China; ^3^MOE Joint International Research Laboratory of Animal Health and Food Safety, College of Veterinary Medicine, Nanjing Agricultural University, Nanjing, China

**Keywords:** TIGIT, *Toxoplasma gondii*, T cells, CD226, T_EM_

## Abstract

As an immunosuppressive receptor, T-cell immunoglobulin and immunoreceptor tyrosine-based inhibitory motif domain (TIGIT) play a critical part in cellular immune regulation mediated by pathogen infection. Whereas, TIGIT expression on splenic T cells in hosts infected with *Toxoplasma gondii* cysts has not been studied. In this study, we detected TIGIT expression and the changes of immune function in the spleen by flow cytometry and real-time PCR (RT-PCR). We found that TIGIT expression on splenic T cells increased significantly post infection. At the same time, splenic TIGIT^+^T_CM_ cells were activated and transformed into TIGIT^+^T_EM_ cells during the infection, and the cytotoxicity of TIGIT^+^ T cells was reduced in the later stage of infection. This study shows that chronic *T. gondii* infection can upregulate TIGIT expression on the surface of T cells and affect immune cell function.

## Introduction

Toxoplasmosis caused by *Toxoplasma gondii* (*T. gondii*) infection has become an important zoonosis in the world, which is often transmitted *via* cysts in raw or under cooked meat or oocysts in cat feces (Fisch et al., 2019). Chronic *T. gondii* infection can lead to the formation of long-term and stable *T. gondii* cysts in multiple host tissues (Pinto-Ferreira et al., 2019). When host immune function is normal, *T. gondii* generally form long-term, stable cysts in infected tissues after 2–3 weeks of acute infection, which continuously stimulates the body to produce an immune response and results in chronic toxoplasmosis (Landrith et al., 2015). *T. gondii* cysts can survive for a long time. However, when the immune function of the host is impaired (such as patients with HIV/AIDS, cancer, or a transplanted organ), bradyzoites in the cyst can escape from the cyst and cause acute infection, resulting in morbidity or death of the host (Montoya and Liesenfeld, 2004; Pinto-Ferreira et al., 2019). At present, there is no ideal drug to control and remove cysts during chronic *T. gondii* infection (Alday and Doggett, 2017). The long-term survival of tissue cysts mainly depends on the effective escape mechanism of *T. gondii* to host cellular immunity, so to explore the mechanism of *T. gondii* causing the failure of host T cells in the process of infection is the fundamental way to effectively removal cysts.

As the main immune organ of T cell immune response against pathogen infection, spleen controls *T. gondii* infection (Zorgi et al., 2016). Meanwhile, an important feature of intracellular pathogenic infection is that it causes the host spleen-specific T cells to proliferate rapidly and secrete a variety of functional cytokines. *T. gondii* infection often causes host T cell proliferation, mediates cytotoxicity and produces cytokines, such as TNF-α and IFN-γ, which plays an important part in anti-*T. gondii* infection (Landrith et al., 2015; Ochiai et al., 2016).

A growing number of studies have proven that high expression of immunosuppressive receptors PD-1 and TIM-3 in host spleen T cells during *T. gondii* infection is related to the inhibition of T cells effector function and reactivation of life-threatening toxoplasmic encephalitis (Bhadra et al., 2012; Wu et al., 2013). It was found that the expression of Tim-3 is positively correlated with IFN-γ, which plays a key part in the protective immunity against *T. gondii* infection (Berrocal Almanza et al., 2013). In addition, blocking PD-1 pathway can significantly restore T cell function and improve the survival rate of mice infected with *T. gondii* (Xiao et al., 2018). Therefore, to determine whether other immunosuppressive receptors are involved in the process of *T. gondii* infection and how they affect infection, it is very important to fully understand the mechanism of T cell immune function exhaustion caused by *T. gondii* infection. Identification of other immunosuppressive receptors is essential for understanding the correlation between T cell depletion and *T. gondii* infection.

TIGIT is a new member of the CD28 family, which can be expressed on almost all T cell subsets (except CD4^+^ naive memory T cells) and NK cells (Solomon and Garrido-Laguna, 2018). TIGIT interacts with CD155 (PVR: poliovirus receptor), CD112 (PVRL2), CD113, and CD226 (DNAM-1) to regulate the immune responses of T cells and NK cells. As the main ligand of TIGIT, CD155 is expressed on the surface of non-hematopoietic cells and is a common ligand shared with the costimulatory molecule CD226. CD226 and TIGIT can competitively bind CD155, with TIGIT inhibiting the activation of T cells, and CD226 promoting the activation of T cells; thus, CD226 and TIGIT play opposite immunological functions and jointly regulate the dynamic balance of human immune function (Bottino et al., 2003; Johnston et al., 2014). Studies have shown that tumors, and viral and parasitic infections can upregulate the expression of TIGIT on T cells in the host spleen, which has a negative correlation with immune function (Johnston et al., 2014; Vendrame et al., 2020; Zhang et al., 2020; Wang et al., 2021). However, how chronic *T. gondii* infection regulates TIGIT expression on splenic T cells and its correlation with T cell function has not been reported.

In this study, our purpose was to study TIGIT expression on splenic T cells and the functional changes in spleen T cells during chronic *T. gondii* infection.

## Materials and Methods

### Mice and Parasites

The PRU strain (type II, low virulence strain) of *T. gondii* used in this study was preserved by the Xinxiang Key Laboratory of Pathogenic Biology, Xinxiang Medical University (Henan, China). The *T. gondii* PRU strain was preserved in C57BL/6 mice by cyst passage. Male C57BL/6 mice (7–8 weeks old) were purchased from Beijing Vital River Experimental Animal Technology Co., Ltd. (Beijing, China) and kept in a specific pathogen-free facility.

### *T. gondii* Infection

To detect the changes in TIGIT expression on and function in splenic T cells post infection, 280 mice were randomly divided into two groups. One group was challenged with 10 PRU cysts by oral administration (infection group), and the other group was treated with a phosphate-buffered saline (PBS) solution (control group).

### Harvest and Preservation of the Spleen

Mice were sacrificed at 0, 1, 3, 6, 9, and 12 weeks post infection (*n* = 10 per group). Mouse spleens were removed aseptically to determine spleen index, and then five spleens were ground into powder by liquid nitrogen and frozen at −80°C for DNA and RNA acquisition. The other five spleens were fixed, sectioned and stained with hematoxylin and eosin (H&E), and then evaluated for the extent of tissue damage.

### Splenic Mononuclear Cells Preparation

Our previous research procedure was applied to obtain splenic mononuclear cells (SMCs; Wang et al., 2021). The spleen was crushed and filtered through 200 mesh nylon net. Then use the lymphocyte separation solution to collect the middle lymphocytes at the interface, the lymphocytes were washed twice, counted and stored.

### Flow Cytometry

SMCs were incubated with FcR Blocking Reagent to block non-specific immunoglobulin binding to Fc receptors, and then cell-surface molecules were stained. Next, the cells were fixed and permeabilized with the FIX&PERM Kit, and further intracellular staining was performed. Cells were incubated with specific antibodies or isotype controls, according to the manufacturer’s guidelines. Antibodies for surface staining were consistent with those reported in our previous research (Wang et al., 2021). The antibodies for intracellular staining antibody were as follows: anti-Granzyme B (anti-human/mouse PE, BioLegend), anti-Perforin (anti-mouse PE, BioLegend), Granzyme B κ Isotype Ctrl (Mouse IgG1 PE, BioLegend), and Perforin κ Isotype Ctrl (Rat IgG2a PE, BioLegend). The isotype controls were used to define positive cells and determine the corresponding gate. Under the same application settings, all flow cytometry samples were detected and analyzed on a CytoFLEX (Beckman Coulter, Brea, CA, United States) with CytExpert 2.1 software.

### Quantitative Real-Time PCR

Total RNA or DNA was extracted from spleens using TRIzol reagent (Yi Fei Xue Biotechnology, Nanjing, China) or a Tissue DNA kit (OMEGA, Zhengzhou, China), and then RNA was converted into first-strand cDNA. The real-time PCR (RT-PCR) was run on a QuantStudio™ 5 (Applied Biosystems, Foster City, CA, United States) with SYBR qPCR Master Mix as described previously (Wang et al., 2021). The primer information used herein is shown in Table 1. The DNA samples were used for the detection of TgB1 gene represents the parasite load, which were normalized for the mouse beta-actin-1 primers (Bhadra et al., 2011). The mRNA expression levels of target genes, such as TIGIT, were normalized to those of the mouse beta-actin-2 primers. Relative expression levels were calculated by the 2^−△△Ct^ method.

**Table 1:** Sequences of the primers used in this study.

Gene	Primer sequence	Target gene length
TgB1		
Sense primer	5ꞌTCCCCTCTGCTGGCGAAAAGT3ꞌ	97 bp
Antisense primer	5ꞌAGCGTTCGTGGTCAACTATCGATTG3ꞌ	
Mouse beta-actin^#^		
Sense primer	5ꞌTCACCCACACTGTGCCCATCTACGA3ꞌ	295 bp
Antisense primer	5ꞌCAGCGGAACCGCTCATTGCCAATGG3ꞌ	
TIGIT		
Sense primer	5ꞌGGCATGTCGCTTCAGTCTTC3ꞌ	139 bp
Antisense primer	5ꞌCTCCCCTTGTAAATCCCACC3ꞌ	
CD226		
Sense primer	5ꞌACCACATGGCTTTCTTGCTC3ꞌ	112 bp
Antisense primer	5ꞌCAGCATGAGAGTTGGACCAG3ꞌ	
IL-2		
Sense primer	5ꞌCAAGCAGGCCACAGAATTGA 3ꞌ	80 bp
Antisense primer	5ꞌGAGTCAAATCCAGAACATGCCG3ꞌ	
IL-12		
Sense primer	5ꞌCTTAGCCAGTCCCGAAACCT3ꞌ	144 bp
Antisense primer	5ꞌACAGGTCTTCAATGTGCTGGT3ꞌ	
IFN-γ		
Sense primer	5ꞌTCAAGTGGCATAGATGTGGAAGAA3ꞌ	267 bp
Antisense primer	5ꞌCTGGACCTGTGGGTTGTTGA3ꞌ	
TNF-α		
Sense primer	5ꞌAGCCGATGGGTTGTACCTTG3ꞌ	99 bp
Antisense primer	5ꞌATAGCAAATCGGCTGACGGT3ꞌ	
Perforin		
Sense primer	5ꞌGAGAAGACCTATCAGGACCA3ꞌ	167 bp
Antisense primer	5ꞌAGCCTGTGGTAAGCATG3ꞌ	
Granzyme B		
Sense primer	5ꞌCCTCCTGCTACTGCTGAC 3ꞌ	174 bp
Antisense primer	5ꞌGTCAGCACAAAGTCCTCTC3ꞌ	
Mouse beta-actin^##^		
Sense primer	5ꞌGATGCAGAAGGAGATTACTG3ꞌ	91 bp
Antisense primer	5ꞌACCGATCCACACAGAGTA3ꞌ	

# *Indicates mouse beta-actin-1 primers in manuscript*.

## *Indicates mouse beta-actin-2 primers in manuscript*.

### Data Analysis

Statistical analysis was performed using SPSS 20 software for Windows (SPSS Inc., Chicago, IL, United States). The differences between the two groups were compared by Student’s *t* test, and those among multiple groups were compared by one-way ANOVA. A value of *p* < 0.05 was considered as statistically significant.

## Results

### Histopathological Changes in the Spleen Were Positively Correlated With the *T. gondii* Parasite Load

**Figure 1:**
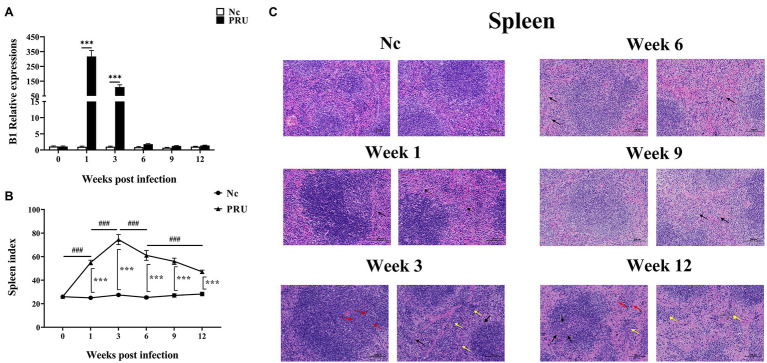
Dynamic pathological changes in the spleen during chronic *Toxoplasma gondii* infection. **(A)** Relative expression of TgB1in the spleen at 0, 1, 3, 6, 9, and 12 weeks post infection in the spleen. **(B)** Spleen index values of the PRU and Nc groups. The results are representative of three independent experiments with five mice per group per experiment; with data denoting means ± SDs; **(C)** H&E staining of spleen sections at different time points post infection. ***^,###^*p* < 0.001, ^*^Indicates comparison with the control group. ^#^Indicates comparison with other time points. The black arrow indicates neutrophil infiltration in the red pulp, the yellow arrow indicates extramedullary hematopoietic cell infiltration, and the red arrow indicates extramedullary hematopoietic cell proliferation.

The relative expression of TgB1 in mice spleens increased sharply beginning in the 1st week post infection, until it was downregulated compared with the control group at the 6th week after infection (Figure 1A). Meanwhile, the spleen of mice rapidly enlarged from 1 week post infection, and the spleen index increased sharply and reached the peak at the 3rd week after infection (Figure 1B). Through H&E staining of spleen sections, we observed that there were no obvious pathological changes in the spleen in the 1st week post infection, only a small number of neutrophils were found in the red pulp, and at the 3rd week after infection, more neutrophils infiltration and a small number of extramedullary hematopoietic cells could be seen in the splenic red pulp. Local lymphocytes in white pulp decreased and extramedullary hematopoietic cells proliferated. At the 6th and 9th week after infection, the spleen returned to normal, with only a small number of neutrophils scattered in the red pulp, and at the 12th week after infection, the red and white pulp of the spleen was clearly demarcated, the number of lymphocytes in the red pulp decreased and the cells arranged loosely, and a small number of extramedullary hematopoietic cells were observed, and extramedullary hematopoietic cells eroded the white pulp, and a small number of neutrophils were scattered in the red pulp (Figure 1C). The results showed that *T. gondii* proliferated in the spleen during the first 3 weeks after infection, resulting in pathological enlargement of the spleen and immune function, thus eliminating *T. gondii*. At the 6th week after infection, *T. gondii* in the spleen was cleared and the spleen returned to normal.

### TIGIT Expression on Splenic T Cells Was Specifically Upregulated by Chronic *T. gondii* Infection

**Figure 2:**
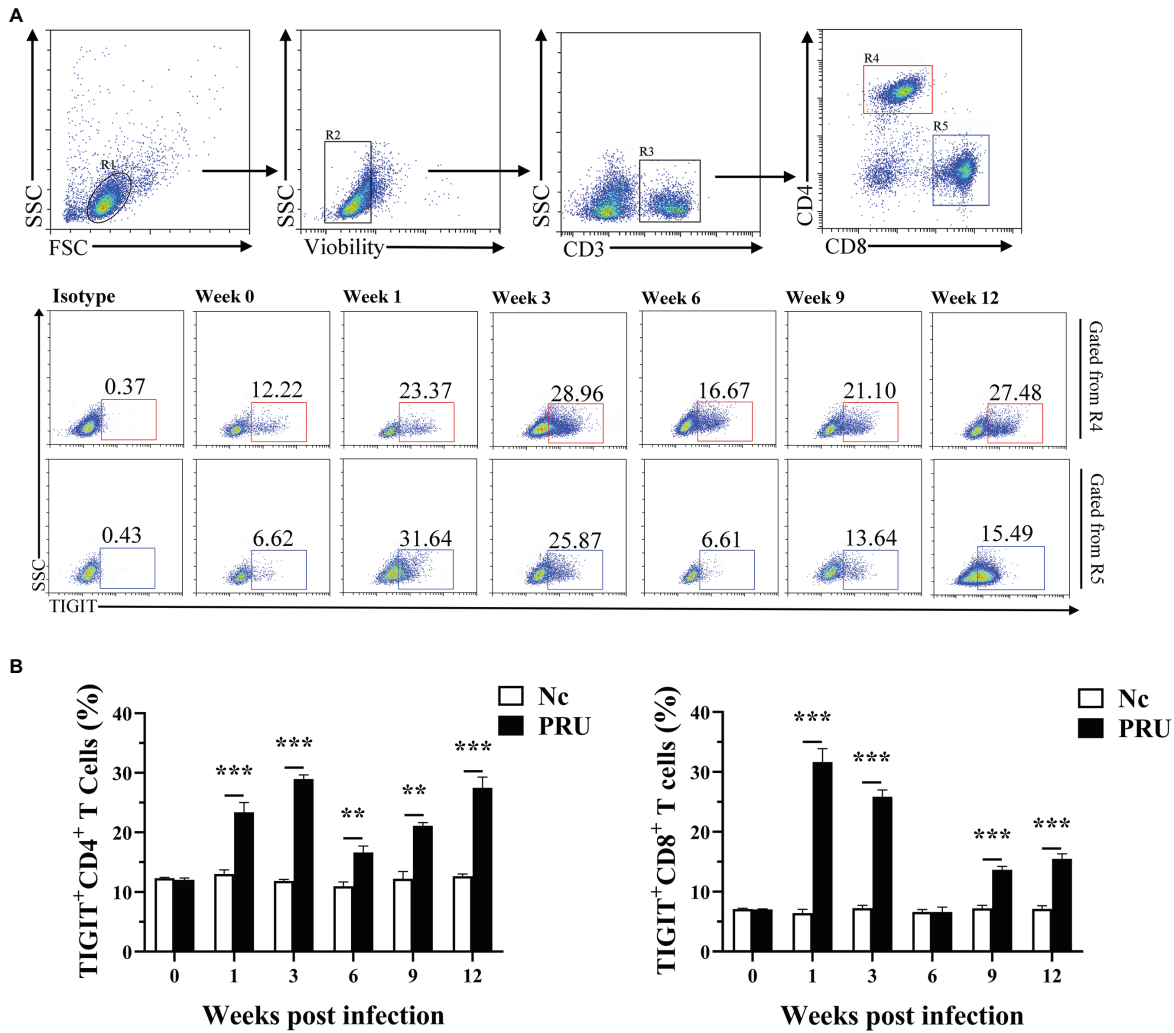
Changes in TIGIT expression on T cells in spleen after *T. gondii* infection. **(A)** Proportions of TIGIT^+^ cells among CD4^+^ and CD8^+^ T cells in *T. gondii*-infected (PRU) and normal mice (Nc) after infection. **(B)** Dynamic changes in the percentages of TIGIT^+^ T cells at different time points. The results are representative of three independent experiments with five mice in each group per experiment, with data denoting means ± SDs. **p < 0.01 and ***p < 0.001 (compared to the control).

As shown in Figure 2, compared with the Nc (normal control) group, TIGIT expression on splenic CD4^+^ T cells of mice was significantly upregulated from the 1st week after PRU cyst infection to the 12th week after infection (*p* < 0.01). Similarly, the proportion of splenic TIGIT^+^CD8^+^ T cells of the infected group did not change significantly only at the 6th week after infection but was higher than that of the Nc group at other time points (*p* < 0.01).

### CD226 Expression on Splenic T Cells Was Specifically Regulated by Chronic *T. gondii* Infection

**Figure 3:**
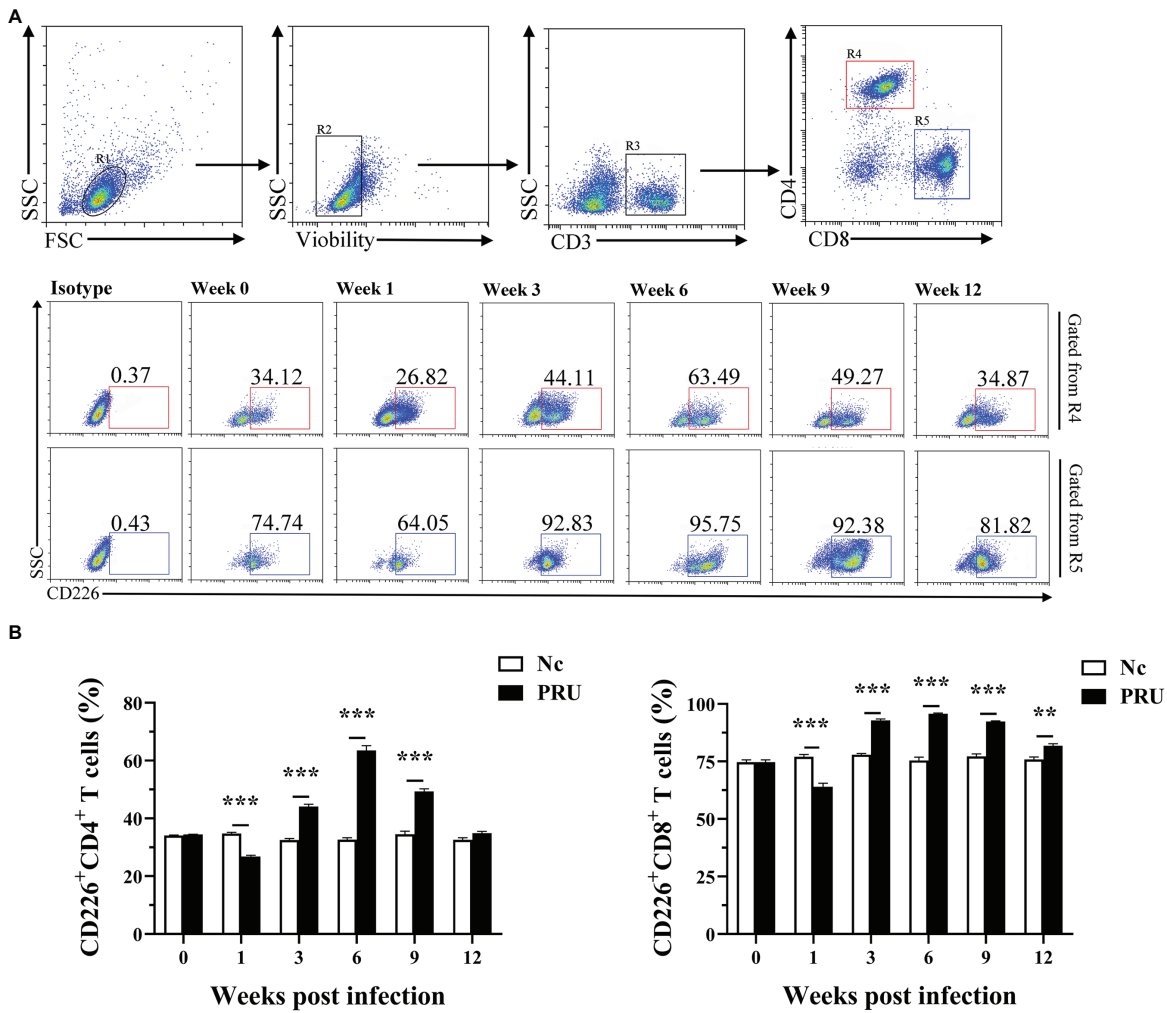
Changes in CD226 expression on T cells in spleen after *T. gondii* infection. **(A)** Proportions of CD226^+^ cells among CD4^+^ and CD8^+^ T cells in *T. gondii*-infected (PRU) and normal mice (Nc) after infection. **(B)** Dynamic changes in the percentages of CD226^+^ T cells at different time points. The results are representative of three independent experiments with five mice in each group per experiment, with data denoting means ± SDs. **p < 0.01 and ***p < 0.001 (compared to the control).

CD226 expression on splenic T cells of mice was significantly downregulated in the 1st week after PRU cyst infection (Figure 3A). In addition, except that there was no prominent change in the proportion of CD226^+^CD4^+^ T cells at the 12th week after infection, CD226 expression on splenic T cells was notably higher than that of the control group since the 3rd week after infection (Figure 3B).

### TIGIT^+^ T_EM_ Cells Were Triggered by Chronic *T. gondii* Infection

**Figure 4:**
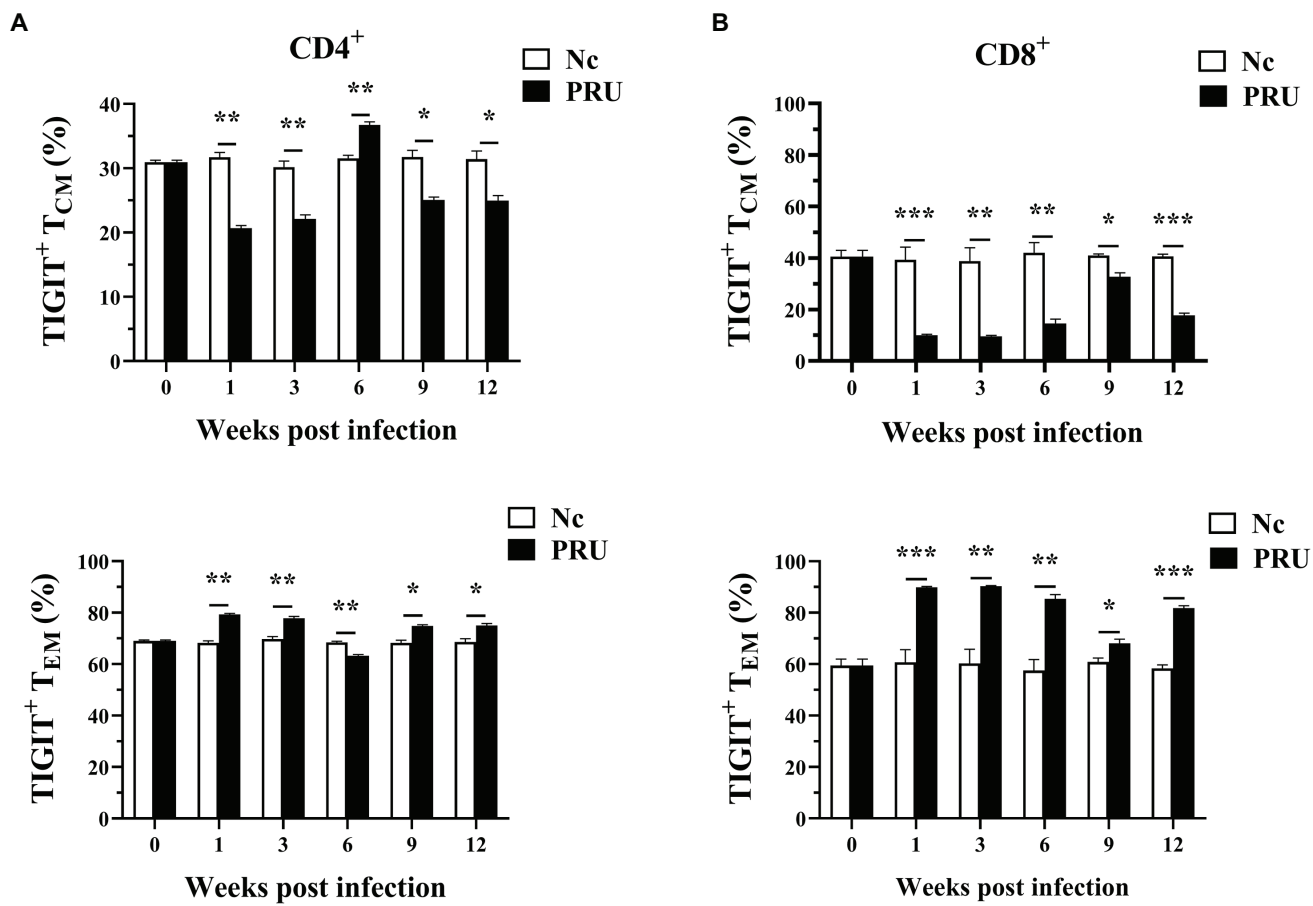
Relative contributions of memory splenic T cell subsets to TIGIT^+^ T cells after *T. gondii* infection. **(A)** Dynamic changes in memory T cell subsets of TIGIT^+^CD4^+^ T cells in the spleen at different time points following PRU infection. **(B)** Dynamic changes in memory T cell subsets of TIGIT^+^CD8^+^ T cells in the spleen at different time points following PRU infection. The results are representative of three independent experiments with five mice in each group per experiment, with data denoting means ± SDs. *p < 0.05, **p < 0.01, and ***p < 0.001 (compared to the control).

Among the memory T cells, T_CM_ (central memory T cells) and T_EM_ (effector memory T cells) are important T cell subsets that play immune-protective roles in the host infected by intracellular pathogens. We labeled the surface of T cells with antibodies of against CD44 and CD62L and classified splenic memory T cells into four subtypes of T_CM_ (CD44^+^CD62L^+^), T_EM_ (CD44^+^CD62L^−^), T_naive_ (CD44^−^CD62L^+^), and T_EMRA_ (CD44^−^CD62L^−^). The subsets of splenic TIGIT^+^ memory T cells were analyzed. As shown in Figure 4, the results were consistent with those of our previous studies on *T. gondii* infection with RH tachyzoites. The specific memory TIGIT^+^ T cell subsets in *T. gondii* infection were mainly T_CM_ and T_EM_ cells. Except for 6 weeks after infection, part of the specific TIGIT^+^CD4^+^ T cells of *T. gondii* in the spleen of the PRU group were activated and transformed into T_EM_. Additionally, *T. gondii* specific memory TIGIT^+^CD8^+^ T cell subsets changed correspondingly at all the time points.

### The Cytotoxicity of TIGIT^+^ T Cells Decreased During Chronic *T. gondii* Infection

**Figure 5:**
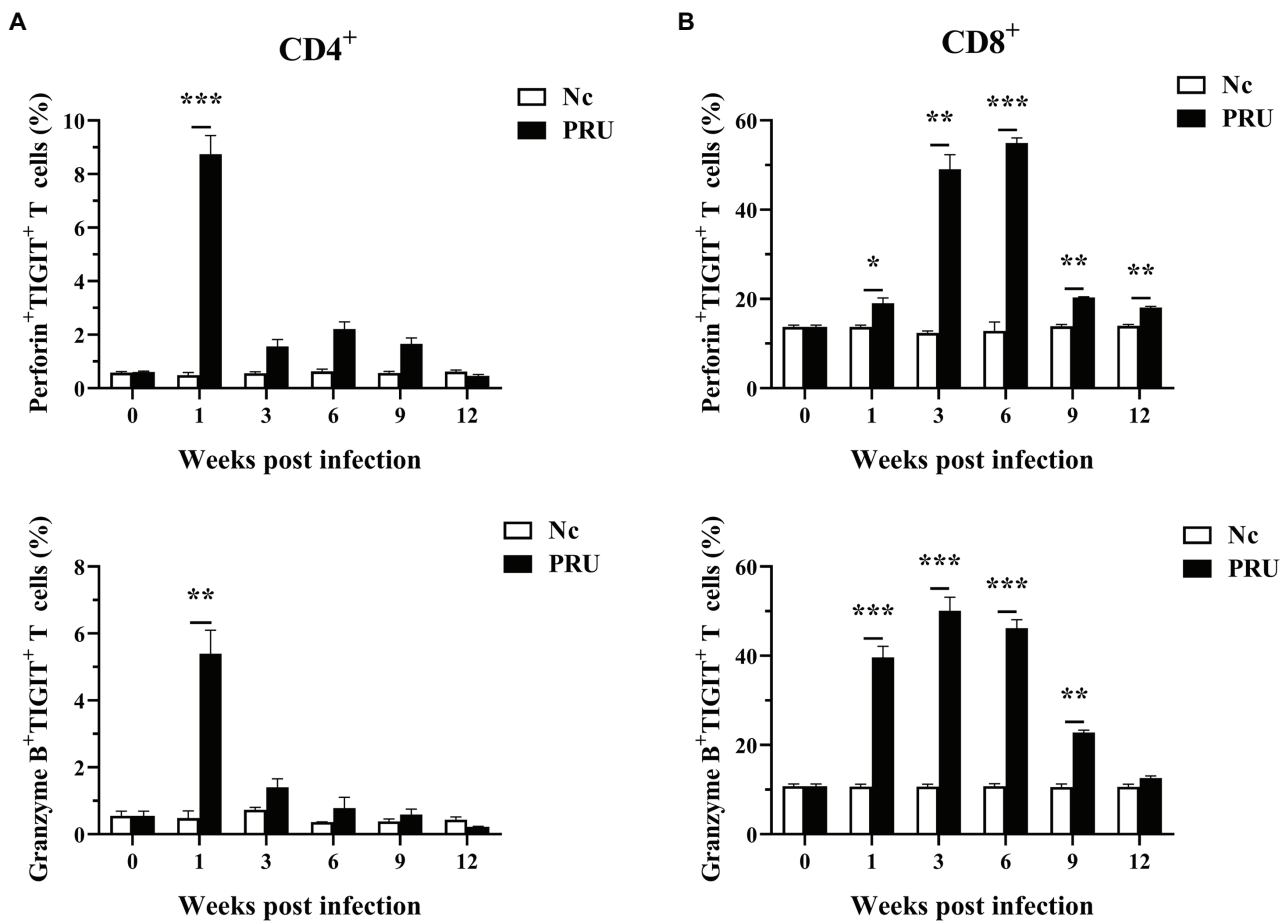
Cytotoxic activity of TIGIT^+^ T cells in spleen after *T. gondii* infection. **(A)** Dynamic changes in TIGIT^+^CD4^+^ T cell cytotoxicity in the spleen of mice chronically infected with *T. gondii*. **(B)** Dynamic changes of TIGIT^+^CD8^+^ T cell cytotoxicity in the spleen of mice chronically infected with *T. gondii*. The results are representative of three independent experiments with five mice in each group per experiment, with data denoting means ± SDs. *p < 0.05, **p < 0.01, and ***p < 0.001 (compared to the control).

As shown in Figure 5, the expression of Perforin (Prf1) and Granzyme B (Gzmb) in the spleen TIGIT^+^ T cells of mice infected with PRU cysts was significantly upregulated at the 1st week post infection (*p* < 0.01). The expression of both Prf1 and Gzmb in TIGIT^+^CD4^+^ T cells decreased from the 3rd week after infection to no significant difference from that in the Nc group, while Prf1 expression in TIGIT^+^CD8^+^ T cells was always higher than that in the Nc group (*p* < 0.05). Gzmb expression in TIGIT^+^CD8^+^ T cells remained higher than that in the control group until it returned to normal level at the 12th week after infection. The results showed that chronic toxoplasmosis is controlled by normal cytotoxicity of host T cells in the early stage, but the cytotoxic effect of TIGIT^+^T cells was weakened with persistent infection.

### TIGIT, CD226, Perforin, Granzyme B, IL-2, IL-12, IFN-γ, and TNF-α mRNA Expression in the Spleen

**Figure 6:**
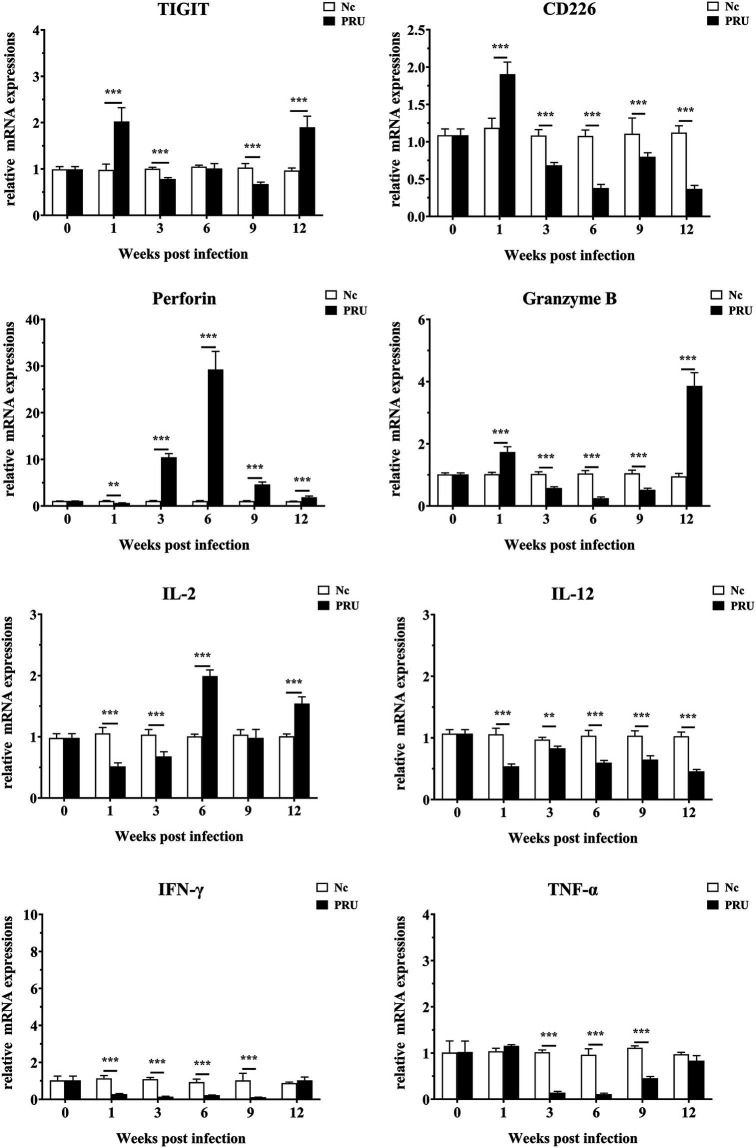
mRNA expression of multiple transcripts in spleens from mice infected with the *T. gondii* PRU strain, as assessed by quantitative real-time PCR (qRT-PCR). Values are shown as the means of triplicate measurements, with data denoting means ± SDs; three independent experiments were performed with five mice per group. **p < 0.01 and ***p < 0.001 (compared to the control).

As shown in Figure 6, compared with those in the control group, TIGIT mRNA expression in spleen was prominently upregulated at the 1st and 12th week post infection (*p* < 0.01), and the mRNA level of TIGIT was prominently downregulated at the 3rd and 9th week post infection (*p* < 0.01). Additionally, CD226 mRNA expression in spleen increased significantly in the 1st week post infection (*p* < 0.01) and decreased significantly at other time points (*p* < 0.01). On the contrary, Prf1 mRNA expression decreased significantly in the 1st week post infection (*p* < 0.01) and increased significantly at other time points (*p* < 0.01). Gzmb mRNA expression in spleen was significantly higher than that in the Nc group only at the 1st and 12th week after infection (*p* < 0.01), and markedly lower than that in the Nc group at other time points (*p* < 0.01). The expression of inflammatory factor IL-2 in spleen decreased significantly from 1 to 3 weeks post infection (*p* < 0.01), and the expression of IL-2 was markedly higher than that in Nc group at 6 and 12 weeks post infection (*p* < 0.01). IL-12 mRNA expression was markedly lower than that in the Nc group (*p* < 0.01) and remained until the 12th week after infection. The expression of IFN-γ was significantly downregulated from 1 to 9 weeks after infection and returned to normal at the 12th week after infection (*p* < 0.01). Furthermore, different from IFN-γ, the expression of TNF-α had no significant difference in the 1st week after infection.

## Discussion

TIGIT can participate in splenic immune responses induced in many chronic infectious diseases (Wykes and Lewin, 2018; Blazkova et al., 2021). Virological studies showed that the proportion of TIGIT^+^CD8^+^ T cells were increased significantly in HIV patients, which was positively correlated with plasma viral load and disease progression. After treatment with an anti-TIGIT monoclonal antibody, the secretion of IFN-γ from HIV-specific NK cells and CD8^+^ T cells was significantly increased. Double blockade of the TIGIT and PD-L1 pathways could significantly improve the ability of T cells to produce IL-2, thus prominently enhancing HIV-specific CD8^+^ T cell proliferation. It is suggested that TIGIT can be used as a potential target for immunotherapy in the treatment of HIV infection (Chew et al., 2016; Yin et al., 2018; Vendrame et al., 2020). Yasuma et al. found that the HBZ protein expressed by human T-cell leukemia virus type 1 (HTLV-1) can induce an increase in TIGIT expression and inhibit the transcription of the CD226 gene, which further enhances the inhibitory function of TIGIT. In addition, a large amount of TIGIT enhances the transcription of IL-10, allowing infected cells to escape being clearance by the immune system (Yasuma et al., 2016). The proportion of TIGIT^+^ T cells in peripheral blood of hepatitis B virus (HBV)-associated hepatocellular carcinoma (HBV-HCC) patients was significantly higher than that in healthy blood donors and hepatitis B virus (HBV)-associated liver cirrhosis (HBV-LC) patients, and the number of TIGIT^+^CD8^+^ T cells was positively correlated with tumor recurrence, tumor invasion and mortality of HBV-HCC, and higher frequency of TIGIT^+^CD8^+^ T cells was more closely related to poor prognosis of HBV-HCC than that of TIGIT^+^CD4^+^ T cells (Liu et al., 2019). In addition, TIGIT on T cell surface was found to be significantly upregulated in patients with lymphocytic choriomeningitis virus (LCMV) or human papilloma virus (HPV) infection (Gameiro et al., 2018; Schorer et al., 2020).

In the field of parasitology, Zhang et al. (2019b) proved that TIGIT expression on the surface of CD4^+^ and CD8^+^ T cells was upregulated in patients with alveolar echinococcosis, and that higher levels of Gzmb were present in TIGIT^−^CD8^+^ T cells rather than in TIGIT^+^CD8^+^ T cells. *Plasmodium yoelii* infection can induce an increase in TIGIT expression on splenic CD4^+^ T cells in mice (Villegas-Mendez et al., 2016). Additionally, blocking the TIM-3 pathway can lead to a compensatory increase in the expression of TIGIT in mice, resulting in the death of infected mice (Zhang et al., 2019a). *Schistosoma japonicum* egg antigen can increase TIGIT expression on CD4^+^ T cells, and the expression of TIGIT can enhance the proliferation of the Th2 cells, thus enhancing Th2 immune response in infected mice (Li-Na et al., 2018). In addition, our previous study showed that selective TIGIT expression on host splenic T cells could be induced by acute infection with *T. gondii* virulent strain (RH) tachyzoites, and that an increase in the proportion of T_EM_ subtypes in TIGIT^+^T cells could be observed (Wang et al., 2021). However, TIGIT expression on splenic immune cells of mice chronically infected with *T. gondii* cysts and its effect on immune function are not clearly characterized.

This study found that, different from our previous studies on acute *T. gondii* infection, *T. gondii* could be detected in the spleen from 1 to 3 weeks post infection, and TIGIT expression on T cells was significantly upregulated. However, with the formation of cysts in the tissue, the pathological sections showed that the spleen returned to normal at the 6th week after infection, and the spleen exerted its immune function and cleared the free *T. gondii* in the spleen. TIGIT expression on splenic CD8^+^ T cells returned to the normal level, but the expression of TIGIT in spleen increased again 9 weeks after infection, which may be related to the partial activation of *T. gondii* in mice. At the same time, compared with the control group, CD226 expression on splenic T cells was significantly downregulated only in the 1st week after infection, which was negatively correlated with the expression of TIGIT. However, with the formation of chronic toxoplasmosis, the immune system is gradually disordered, the mutual antagonism between them disappears, and the expression on T cells is significantly upregulated, which may be due to the existence of other modes of action between TIGIT and CD226 expressed during *T. gondii* infection, which need to be further studied.

The relative changes in T cell subsets in patients with pathogen infection affect T cell immune function and disease occurrence and development (Schlüter et al., 2002; Mueller et al., 2013). Research has shown that high expression of TIGIT in memory cell subsets of CD4^+^ T cells in patients with acute HCV infection, and the expression of TIGIT was the highest in effector memory T cells and the lowest in initial memory T cells, but TIGIT expression was relatively stable in T_CM_ and T_EMRA_ subsets (Ackermann et al., 2019). Therefore, we analyzed the changes of the proportions of specific TIGIT^+^ T cell subsets during *T. gondii* cysts infection by evaluating CD44 and CD62L. Similar to our previous studies, TIGIT^+^ T cells in mouse spleen were mainly divided into T_CM_ and T_EM_ after *T. gondii* cysts infection. Furthermore, studies have shown that continuous antigen stimulation can lead to the activation of T_CM_ cells to produce T_EM_ subsets during chronic parasite infection, and that long-term antigen stimulation keeps the working intensity of memory T cells at a high level, resulting in T cell functional exhaustion. In this study, spleen CD8^+^TIGIT^+^ T_CM_ cells was activated and transformed into CD8^+^TIGIT^+^ T_EM_ cells during infection. Similarly, except for the 6th week after infection, CD4^+^TIGIT^+^ T cells also had the same changes, indicating that *T. gondii* infection stimulated the host TIGIT^+^ T_EM_ cells to proliferate, migrate to the inflammatory surrounding tissue, and produce cytokines to control *T. gondii* infection.

Prf1 and Gzmb, as the main factors mediating the cytotoxicity of T cells, can kill infected cells from in a host and have an important effect on T cells defending against *T. gondii* infection (Yamada et al., 2011; Suzuki, 2020). In addition, the perforin dependent cytotoxic ability of T cell is involved in restricting the parasite to chronic state (Suzuki et al., 2010). Therefore, we detected the expression of Prf1 and Gzmb in TIGIT^+^ T cells. The results showed that cytotoxicity of splenic TIGIT^+^ T cells increased prominently in the early-stage infection of *T. gondii* cysts, but decreased in varying degrees after the stable existence of *T. gondii* cysts, indicating that the change of cytotoxicity of splenic TIGIT^+^ T cells may be related to the formation and rupture of *T. gondii* cysts.

We further detected the changes in TIGIT gene transcriptional expression at the overall level in spleen during infection and found that it was significantly up-regulated in the 1st week after infection, then downregulated, and upregulated again in the 12th week after infection, when the spleen sections also showed abnormal pathological changes. At the same time, Gzmb also showed the same dynamic expression changes as TIGIT. However, the expressions of IL-12, IFN-γ, and TNF-α were not upregulated, suggesting the immune function has not been brought into full play. However, the effect of TIGIT on the proliferative activity of *T. gondii* specific T cells and its expression on other immune cells such as Treg and NK cells during *T. gondii* infection are not clear, and the changes of immune function of host splenic T cells by blocking the TIGIT pathway need to be further investigated.

## Conclusion

This study shows that chronic infection of *T. gondii* cysts can increase the TIGIT expression in host splenic T cells, stimulate the host TIGIT^+^ T_EM_ cells to proliferate, and weaken the cytotoxicity of TIGIT^+^ T cells. Therefore, TIGIT is expected to be a therapeutic target for chronic *T. gondii* infection and provides new insights into prevention and treatment of *T. gondii* infection.

## Data Availability Statement

The raw data supporting the conclusions of this article will be made available by the authors, without undue reservation, to any qualified researcher.

## Ethics Statement

All animal experiments were reviewed and approved by the Ethics Committee of Xinxiang Medical University.

## Author Contributions

SW, XL, and MW: conceptualization and methodology. HL, CS, and JZ: formal analysis and investigation. HL, XT, and JZ: data curation. HL, JZ, XT, and XM: data curation. HL: writing – original draft preparation. ZZ, MW, XL, and SW: writing – review and editing. MW, XL, and SW: funding acquisition. All authors contributed to the article and approved the submitted version.

## Conflict of Interest

The authors declare that the research was conducted in the absence of any commercial or financial relationships that could be construed as a potential conflict of interest.

## Publisher’s Note

All claims expressed in this article are solely those of the authors and do not necessarily represent those of their affiliated organizations, or those of the publisher, the editors and the reviewers. Any product that may be evaluated in this article, or claim that may be made by its manufacturer, is not guaranteed or endorsed by the publisher.

## References

[ref1] Ackermann C. Smits M. Woost R. Eberhard J. M. Peine S. Kummer S.. (2019). HCV-specific CD4^+^ T cells of patients with acute and chronic HCV infection display high expression of TIGIT and other co-inhibitory molecules. Sci. Rep. 9:10624. 10.1038/s41598-019-47024-8, PMID: 31337800 PMC6650447

[ref2] Alday P. H. Doggett J. S. (2017). Drugs in development for toxoplasmosis: advances, challenges, and current status. Drug Des. Devel. Ther. 11, 273–293. 10.2147/dddt.S60973, PMID: 28182168 PMC5279849

[ref3] Berrocal Almanza L. C. Muñoz M. Kühl A. A. Kamradt T. Heimesaat M. M. Liesenfeld O. (2013). Tim-3 is differently expressed in genetically susceptible C57BL/6 and resistant BALB/c mice during oral infection with *Toxoplasma gondii*. Eur. J. Microbiol. Immunol. 3, 211–221. 10.1556/EuJMI.3.2013.3.10, PMID: 24265941 PMC3832097

[ref4] Bhadra R. Gigley J. P. Khan I. A. (2012). PD-1-mediated attrition of polyfunctional memory CD8^+^ T cells in chronic toxoplasma infection. J. Infect. Dis. 206, 125–134. 10.1093/infdis/jis304, PMID: 22539813 PMC3415930

[ref5] Bhadra R. Gigley J. P. Weiss L. M. Khan I. A. (2011). Control of *Toxoplasma* reactivation by rescue of dysfunctional CD8^+^ T-cell response via PD-1-PDL-1 blockade. Proc. Natl. Acad. Sci. U. S. A. 108, 9196–9201. 10.1073/pnas.1015298108, PMID: 21576466 PMC3107287

[ref6] Blazkova J. Huiting E. D. Boddapati A. K. Shi V. Whitehead E. J. Justement J. S.. (2021). TIGIT expression on CD8^+^ T cells correlates with higher cytotoxic capacity. J. Infect. Dis. jiab155. 10.1093/infdis/jiab155 [Epub ahead of print], PMID: 33744939 PMC8599894

[ref7] Bottino C. Castriconi R. Pende D. Rivera P. Nanni M. Carnemolla B.. (2003). Identification of PVR (CD155) and nectin-2 (CD112) as cell surface ligands for the human DNAM-1 (CD226) activating molecule. J. Exp. Med. 198, 557–567. 10.1084/jem.20030788, PMID: 12913096 PMC2194180

[ref8] Chew G. M. Fujita T. Webb G. M. Burwitz B. J. Wu H. L. Reed J. S.. (2016). TIGIT marks exhausted T cells, correlates with disease progression, and serves as a target for immune restoration in HIV and SIV infection. PLoS Pathog. 12:e1005349. 10.1371/journal.ppat.1005349, PMID: 26741490 PMC4704737

[ref9] Fisch D. Clough B. Frickel E. M. (2019). Human immunity to *Toxoplasma gondii*. PLoS Pathog. 15:e1008097. 10.1371/journal.ppat.1008097, PMID: 31830133 PMC6907746

[ref10] Gameiro S. F. Ghasemi F. Barrett J. W. Koropatnick J. Nichols A. C. Mymryk J. S.. (2018). Treatment-naïve HPV+ head and neck cancers display a T-cell-inflamed phenotype distinct from their HPV- counterparts that has implications for immunotherapy. Onco. Targets. Ther. 7:e1498439. 10.1080/2162402X.2018.1498439, PMID: 30288365 PMC6169583

[ref11] Johnston R. J. Comps-Agrar L. Hackney J. Yu X. Huseni M. Yang Y.. (2014). The immunoreceptor TIGIT regulates antitumor and antiviral CD8^+^ T cell effector function. Cancer Cell 26, 923–937. 10.1016/j.ccell.2014.10.018, PMID: 25465800

[ref12] Landrith T. A. Harris T. H. Wilson E. H. (2015). Characteristics and critical function of CD8+ T cells in the *Toxoplasma*-infected brain. Semin. Immunopathol. 37, 261–270. 10.1007/s00281-015-0487-3, PMID: 25898888 PMC5313077

[ref13] Li-Na Z. Xiao-Fan W. Qian-Qian Q. Li-Yang D. Lei X. Ya-Nan P.. (2018). Study on role of TIGIT signal in Th1/Th2 balance in *Schistosoma japonicum*-infected mice. Zhongguo Xue Xi Chong Bing Fang Zhi Za Zhi 30, 136–139. 10.16250/j.32.1374.2017233, PMID: 29770653

[ref14] Liu X. Li M. Wang X. Dang Z. Jiang Y. Wang X.. (2019). PD-1^+^ TIGIT^+^ CD8^+^ T cells are associated with pathogenesis and progression of patients with hepatitis B virus-related hepatocellular carcinoma. Cancer Immunol. Immunother. 68, 2041–2054. 10.1007/s00262-019-02426-5, PMID: 31720814 PMC11028102

[ref15] Montoya J. G. Liesenfeld O. (2004). Toxoplasmosis. Lancet 363, 1965–1976. 10.1016/S0140-6736(04)16412-X, PMID: 15194258

[ref16] Mueller S. N. Gebhardt T. Carbone F. R. Heath W. R. (2013). Memory T cell subsets, migration patterns, and tissue residence. Annu. Rev. Immunol. 31, 137–161. 10.1146/annurev-immunol-032712-095954, PMID: 23215646

[ref17] Ochiai E. Sa Q. Perkins S. Grigg M. E. Suzuki Y. (2016). CD8^+^ T cells remove cysts of *Toxoplasma gondii* from the brain mostly by recognizing epitopes commonly expressed by or cross-reactive between type II and type III strains of the parasite. Microbes Infect. 18, 517–522. 10.1016/j.micinf.2016.03.013, PMID: 27083473 PMC4927374

[ref18] Pinto-Ferreira F. Caldart E. T. Pasquali A. K. S. Mitsuka-Breganó R. Freire R. L. Navarro I. T. (2019). Patterns of transmission and sources of infection in outbreaks of human toxoplasmosis. Emerg. Infect. Dis. 25, 2177–2182. 10.3201/eid2512.181565, PMID: 31742524 PMC6874273

[ref19] Schlüter D. Meyer T. Kwok L. Y. Montesinos-Rongen M. Lütjen S. Strack A.. (2002). Phenotype and regulation of persistent intracerebral T cells in murine *Toxoplasma encephalitis*. J. Immunol. 169, 315–322. 10.4049/jimmunol.169.1.315, PMID: 12077260

[ref20] Schorer M. Rakebrandt N. Lambert K. Hunziker A. Pallmer K. Oxenius A.. (2020). TIGIT limits immune pathology during viral infections. Nat. Commun. 11:1288. 10.1038/s41467-020-15025-1, PMID: 32152316 PMC7062903

[ref21] Solomon B. L. Garrido-Laguna I. (2018). TIGIT: a novel immunotherapy target moving from bench to bedside. Cancer Immunol. Immunother. 67, 1659–1667. 10.1007/s00262-018-2246-5, PMID: 30232519 PMC11028339

[ref22] Suzuki Y. (2020). The immune system utilizes two distinct effector mechanisms of T cells depending on two different life cycle stages of a single pathogen, *Toxoplasma gondii*, to control its cerebral infection. Parasitol. Int. 76:102030. 10.1016/j.parint.2019.102030, PMID: 31778800 PMC7136146

[ref23] Suzuki Y. Wang X. Jortner B. S. Payne L. Ni Y. Michie S. A.. (2010). Removal of *Toxoplasma gondii* cysts from the brain by perforin-mediated activity of CD8+ T cells. Am. J. Pathol. 176, 1607–1613. 10.2353/ajpath.2010.090825, PMID: 20167872 PMC2843452

[ref24] Vendrame E. Seiler C. Ranganath T. Zhao N. Q. Vergara R. Alary M.. (2020). TIGIT is upregulated by HIV-1 infection and marks a highly functional adaptive and mature subset of natural killer cells. AIDS 34, 801–813. 10.1097/QAD.0000000000002488, PMID: 32028328 PMC7148170

[ref25] Villegas-Mendez A. Inkson C. A. Shaw T. N. Strangward P. Couper K. N. (2016). Long-lived CD4^+^IFN-γ^+^ T cells rather than short-lived CD4^+^IFN-γ^+^IL-10^+^ T cells initiate rapid IL-10 production to suppress anamnestic T cell responses during secondary malaria infection. J. Immunol. 197, 3152–3164. 10.4049/jimmunol.1600968, PMID: 27630165 PMC5055201

[ref26] Wang S. Li H. Zhang F. Jiao Y. Xie Q. Zhang Z.. (2021). Expression of TIGIT in splenic and circulatory T cells from mice acutely infected with *Toxoplasma gondii*. Parasite 28:13. 10.1051/parasite/2021010, PMID: 33629951 PMC7906093

[ref27] Wu B. Huang B. Chen Y. Li S. Yan J. Zheng H.. (2013). Upregulated expression of Tim-3 involved in the process of Toxoplasmic encephalitis in mouse model. Parasitol. Res. 112, 2511–2521. 10.1007/s00436-013-3416-1, PMID: 23595213

[ref28] Wykes M. N. Lewin S. R. (2018). Immune checkpoint blockade in infectious diseases. Nat. Rev. Immunol. 18, 91–104. 10.1038/nri.2017.112, PMID: 28990586 PMC5991909

[ref29] Xiao J. Li Y. Yolken R. H. Viscidi R. P. (2018). PD-1 immune checkpoint blockade promotes brain leukocyte infiltration and diminishes cyst burden in a mouse model of *Toxoplasma* infection. J. Neuroimmunol. 319, 55–62. 10.1016/j.jneuroim.2018.03.013, PMID: 29685290

[ref30] Yamada T. Tomita T. Weiss L. M. Orlofsky A. (2011). *Toxoplasma gondii* inhibits granzyme B-mediated apoptosis by the inhibition of granzyme B function in host cells. Int. J. Parasitol. 41, 595–607. 10.1016/j.ijpara.2010.11.012, PMID: 21329693 PMC3116727

[ref31] Yasuma K. Yasunaga J. Takemoto K. Sugata K. Mitobe Y. Takenouchi N.. (2016). HTLV-1 bZIP factor impairs anti-viral immunity by inducing co-inhibitory molecule, T cell immunoglobulin and ITIM domain (TIGIT). PLoS Pathog. 12:e1005372. 10.1371/journal.ppat.1005372, PMID: 26735971 PMC4703212

[ref32] Yin X. Liu T. Wang Z. Ma M. Lei J. Zhang Z.. (2018). Expression of the inhibitory receptor TIGIT is up-regulated specifically on NK cells with CD226 activating receptor from HIV-infected individuals. Front. Immunol. 9:2341. 10.3389/fimmu.2018.02341, PMID: 30364127 PMC6192288

[ref33] Zhang Y. Jiang N. Zhang T. Chen R. Feng Y. Sang X.. (2019a). Tim-3 signaling blockade with α-lactose induces compensatory TIGIT expression in *Plasmodium berghei* ANKA-infected mice. Parasit. Vectors 12:534. 10.1186/s13071-019-3788-x, PMID: 31711531 PMC6849286

[ref34] Zhang C. Lin R. Li Z. Yang S. Bi X. Wang H.. (2020). Immune exhaustion of T cells in alveolar echinococcosis patients and its reversal by blocking checkpoint receptor TIGIT in a murine model. Hepatology 71, 1297–1315. 10.1002/hep.30896, PMID: 31410870

[ref35] Zhang C. Shao Y. Yang S. Bi X. Li L. Wang H.. (2019b). Author correction: T-cell tolerance and exhaustion in the clearance of *Echinococcus multilocularis*: role of inoculum size in a quantitative hepatic experimental model. Sci. Rep. 9:3424. 10.1038/s41598-019-39975-9, PMID: 30809024 PMC6391374

[ref36] Zorgi N. E. Galisteo A. J. Jr. Sato M. N. do Nascimento N. de Andrade H. F. Jr. (2016). Immunity in the spleen and blood of mice immunized with irradiated *Toxoplasma gondii* tachyzoites. Med. Microbiol. Immunol. 205, 297–314. 10.1007/s00430-015-0447-5, PMID: 26732075

